# A short deletion in the DNA-binding domain of STAT3 suppresses growth and progression of colon cancer cells

**DOI:** 10.18632/aging.202439

**Published:** 2021-02-01

**Authors:** Yi-Jia Xiong, Dong-Yang Liu, Rong-Rong Shen, Yong Xiong

**Affiliations:** 1 Department of Radiology, Shanghai Jiao Tong University Affiliated Sixth People’s Hospital, Shanghai 200233, China; 2 Department of General Surgery, Shanghai Jiao Tong University Affiliated Sixth People’s Hospital, Shanghai 200233, China; 3 Department of General Surgery, Shanghai Sixth People’s Hospital East Affiliated to Shanghai University of Medicine and Health Sciences, Shanghai 201308, China

**Keywords:** STAT3, gain of function, JAK/STAT pathway, colon cancer

## Abstract

In this study, we investigated the effect of a short deletion in the DNA-binding domain of STAT3 (STAT3^del^) on the transcriptional activation of STAT3 target genes and its relationship with colon carcinogenesis. We used the CRISPR-CAS9 gene editing system to delete a short sequence encoding amino acids 400-411 in the DNA-binding domain (amino acid sequence: 317-567) from *STAT3* gene in SW480, SW620 and HCT116 colon cancer cells. ChIP sequencing analysis showed that STAT3^del^ occupancy was significantly reduced in 1029 genes and significantly increased in 475 genes compared to wild-type STAT3. The mutation altered the DNA motifs recognized by STAT3^del^ as compared to the wild-type STAT3. We observed a strong correlation between expression of the STAT3 target genes and the loss or gain of STAT3^del^ binding to their promoters. CCK-8, wound healing, and TUNEL assays showed reduced proliferation, migration, and survival of SW480, SW620 and HCT-116 cells expressing STAT3^del^ as compared to the corresponding controls. These findings demonstrate that a short deletion in the DNA-binding domain of STAT3 alters its genome-wide DNA-binding and transcriptional profile of STAT3-target proteins, and suppresses the growth, progression and survival of colon cancer cells.

## INTRODUCTION

Colorectal cancer (CRC) is the third most commonly diagnosed cancer [1] that accounts annually for nearly 0.7 million deaths and 1.4 million newly diagnosed cases worldwide [2]. Next-generation sequencing and multi-omics studies have greatly improved our understanding of genes and pathways that regulate cancer development and progression. The Cancer Genome Atlas (TCGA) project was launched to screen genetic alterations in large cohorts of cancer patients through high-throughput genomic sequencing and integrated multi-dimensional analysis, and subsequently identify reliable diagnostic and prognostic biomarkers [3]. In general, these biomarkers are differentially expressed in the tumor tissues compared to para-cancerous or normal tissues.

The signal transducer and activator of transcription (STAT) proteins are an integral component of the Janus activated kinase (JAK)-STAT signaling pathway. JAKs phosphorylate STAT proteins, which enter the nucleus as dimers, bind to the target genes, and activate transcription [4]. STATs contain N-terminal, DNA binding, Src homology 2 (SH2), and C-terminal domains. The N-terminal domain is required for dimer stability, whereas the highly conserved SH2 domain contains the tyrosine residue, which is phosphorylated by JAKs and is required for dimerization [5]. All STAT proteins except STAT2 also contain a putative DNA-binding domain between amino acid residues 400 and 500 of the nearly 750 amino-acid long STAT proteins. However, sequences of different STAT proteins do not show significant homology, thereby suggesting that the STAT protein may target different genes because of variations in DNA motif recognition and affinity [6, 7].

STAT3 is a member of STAT family that is up-regulated in several human malignancies, and is a potential target for anticancer drugs. The crystal structure of the human STAT3-DNA complex suggests that the amino-acid residues between 325 and 464 form the DNA-binding domain [8]. In hyper-IgE syndrome patients, mutations in R382 affect hydrogen-bond interactions with V463 and E435 and disrupt DNA recognition [9]. Moreover, F384, T389, H437 and N466 amino acid residues are all required for STAT3-DNA interface stability. Although the structure of STAT3 is known, the molecular details of changes in DNA-binding properties of mutated STAT3 and their relationship with human diseases including cancers is not clear. Therefore, in this study, we deleted a sequence encoding amino acids 400-411 in the DNA-binding domain of STAT3 (STAT3^del^) using the CRISPR-CAS9 gene editing system in multiple colon cancer cell lines (SW480, SW620, and HCT116) and characterized its effects on genome-wide binding to STAT3-target genes using ChIP-seq analysis. We also performed *in vitro* functional experiments to determine the effects of STAT3^del^ on colon cancer cell growth, progression and survival.

## RESULTS

### The mutation landscape of STAT3 coding region in human cancers

**Figure 1:**
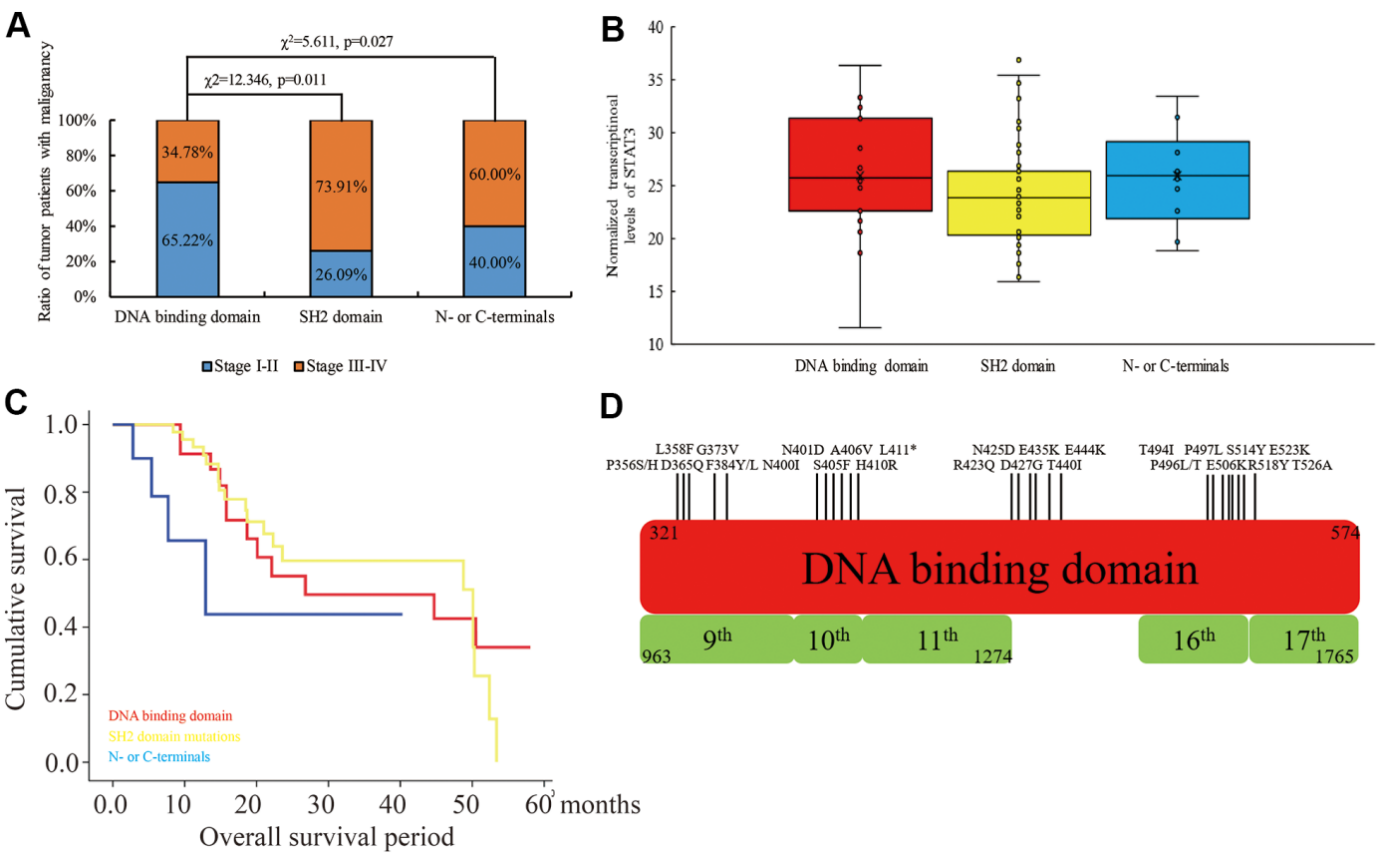
**The relationship between mutations in different domains of *STAT3* and tumor stages, STAT3 mRNA levels and overall survival times of cancer patients belonging to 32 different human cancers in the TCGA database.** (**A**) The ratio of human cancer patients with early (stages I-II; blue) and advanced (stages III-IV; orange) stage cancers in the TCGA database grouped according to the mutations in the DNA-binding domain, SH2 domain and N- or C-terminal regions of the *STAT3* gene. χ^2^ analysis was used to evaluate differences between the groups. (**B**) The levels of STAT3 transcripts in human cancers with mutations in different regions of *STAT3*. (**C**) Kaplan-Meier survival curve analysis shows overall survival of cancer patients from the TCGA database with mutations in the DNA-binding domain (red), SH2 domain (yellow), and N- or C-terminals (blue) of STAT3. (**D**) The overview of the mutational landscape in the DNA-binding domain of the *STAT3* gene.

We used the TCGA database to investigate the mutation status in the coding region of *STAT3* among 10953 cases from 32 types of cancers and identified 1.3% mutation (121 mis-sense and 22 truncating mutations). Among these, we identified 53 mutations in the DNA-binding domain (325-574), 31 mutations in the SH2 domain (584 - 674), and the remaining 73 mutations in other coding regions. We further observed that tumor malignancy was significantly lower in patients with mutations in the DNA-binding domain of *STAT3* compared to those with the mutations in the SH2 domain (χ2=12.346, p=0.011) and the N- or C-terminal regions (χ2=5.611, p=0.027) (Figure 1A). Moreover, STAT3 mRNA expression levels were not associated with *STAT3* gene mutations (p>0.05) (Figure 1B). Furthermore, patients with mutations in the DNA-binding domain (p=0.037) and SH2 domain (p=0.042) of *STAT3* showed significantly higher overall survival rate compared those with mutations in the other regions of the *STAT3* gene (Figure 1C). Overall, mutations were evenly distributed in the DNA-binding domain, but, in more than 50% of cancer types that were derived from epithelioid cells, such as colon, bladder, and uterine endometrioid cancers, the mutations were concentrated in the region between amino acid residues 400 and 411 (“NNGSLSAEFKHL”) compared to the other regions (Figure 1D). Hence, we considered this sequence as a hot spot for mutations in colon cancer and focused on further characterizing the significance of mutation in this region to colon carcinogenesis. Taken together, our results suggest that *STAT3* mutations in the DNA-binding domain are tumor suppressive.

### The genomic binding pattern of mutant STAT3 in colon cancer cells

**Figure 2:**
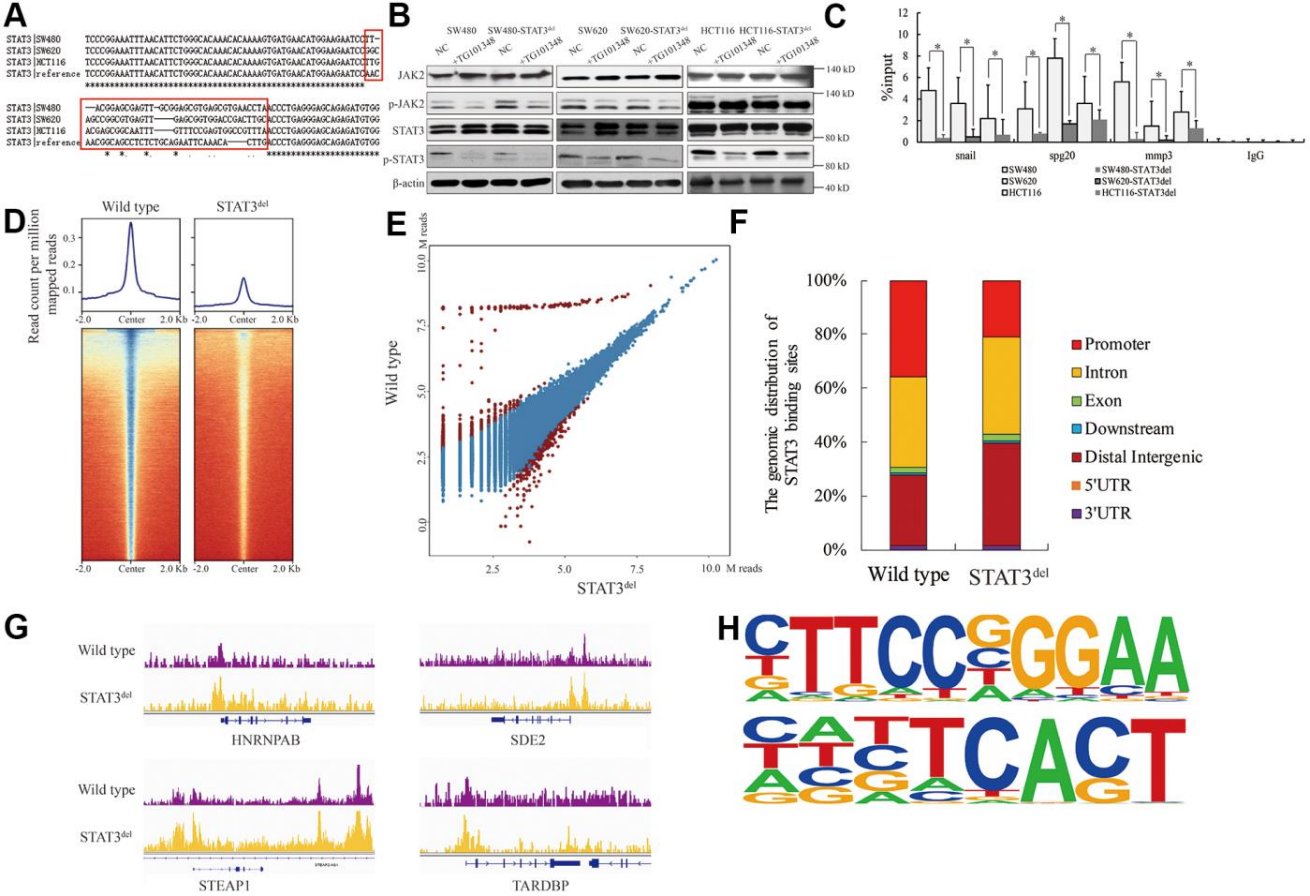
**The genomic binding pattern of STAT3^del^ in colon cancer cells.** (**A**) Sequence alignment shows the deletion of sequence encoding amino acid residues between 400 and 411 (NNGSLSAEFKHL) in the DNA-binding domain of *STAT3* in the colon cancer cells (SW480, SW620, and HCT116) using CRISPR-CAS9. (**B**) Representative western blot shows the total and phosphorylated levels of JAK2 and STAT3 in control and TG101348 (JAK2 inhibitor)-treated wild-type and STAT3^del^ SW480, SW620, and HCT116 cells. Βeta-actin is shown as the loading control. Each experiment was performed in triplicates. (**C**) ChIP-qPCR results show the occupancy of STAT3^del^ in the promoter regions of *Snail*, *Spg20* and *MMP*3 genes in SW480, SW620, and HCT116 cells with wild-type STAT3 or STAT3^del^. * denotes *p* < 0.05. Each experiment was performed in triplicate. (**D**) The heatmap shows the genome-wide occupancy of STAT3 and STAT3^del^ at all the annotated gene promoters in the SW480 cells as determined by ChIP-seq. The average STAT3 enrichment is measured based on the log2 (peak *p* values) in 200-bp bins and is shown within genomic regions covering 2 kb upstream and downstream of the center. (**E**) The volcano plot shows the comparison of different peaks (representing STAT3 genomic occupancy) between wild type and STAT3^del^ SW480 cells. Brown spots represent significantly altered peaks between STAT3 and STAT3^del^; blue spots represent peaks without statistical significance. (**F**) The genome-wide distribution of STAT3 binding regions in wild-type and STAT3^del^ SW480 cells. The various genomic regions are represented by different colors. (**G**) Enrichment of STAT3 (purple) or STAT3^del^ (yellow) in the *HNRNPAB*, *SDE2*, *STEAP1*, and *TARDBP* genes. ChIP-seq data are shown in reads per million; y-axis floor is set to 0.5 reads per million. (**H**) Predicted DNA-binding motifs that are enriched in the loci bound by wild type STAT3 (top) and STAT3^del^ (bottom) in the SW480 cells.

We used the CRISPR-CAS9 gene editing system to delete the sequence encoding “NNGSLSAEFKHL” amino-acid sequence in the *STAT3* gene in SW480, SW620 and HCT116 colon cancer cells and designated it as STAT3^del^. We confirmed the edited genomic sequence by Sanger sequencing (Figure 2A). The activation of STAT3 was independent of the deletion and its phosphorylation by JAK was inhibited by TG101348 (JAK2 inhibitor) treatment (Figure 2B). ChIP-qPCR analysis showed that the DNA-binding affinity of STAT3 was significantly reduced at the known target genes such as *Snail* [10], *Spg20* [11], *Mmp3* [12] in the colon cancer cells with STAT3^del^, especially in the SW480 cells with STAT3^del^ (Figure 2C). We further investigated the alteration in the genome-wide binding pattern of STAT3^del^ in the SW480 cells using ChIP-seq. The raw data consisted of approximately 81.6 M reads from the wild type SW480 cells and 83.2 M reads from the SW480 cells with STAT3^del^ (Supplementary Table 2). The genomic occupancy of STAT3^del^ was significantly lower compared to the wild type STAT3 in SW480 cells (p=0.013; Figure 2D). SW480 cells with STAT3^del^ lost 1029 peaks and gained 475 peaks compared to the wild type SW480 cells (log_2_FC>1 or <-1 and p<0.05; Figure 2E). As shown in Figure 2F, STAT3^del^ enrichment was significantly lower than the wild type STAT3 at the promoter regions (21.3% vs. 35.9%, p=0.024), but significantly higher in the distal intergenic regions (38.3% vs. 26%, p=0.033). The STAT3 and STAT3^del^ binding status in the *HNRNPAB, SDE2, STEAP1* and *TARDBP* genes in the wild type and mutant SW480 is shown in Figure 2G. DNA motif analysis showed that STAT3^del^ recognized a variable DNA motif compared to the “CTTCCGGGAA” motif that is recognized by the wild-type STAT3 (p<0.0001; Figure 2H). The altered DNA recognition motif “HNBTCACT” (symbols for mix-bases) (p<0.0001) was observed in the promoter region of STAT3 target genes such as *HNRNPAB, SDE2, STEAP1* and *TARDBP* genes. These data demonstrate that deletion of the 400-411 amino acid sequence in STAT3 significantly alters its genome-wide binding pattern.

### The tumor suppressive role of the hot spot mutation in the DNA-binding domain of STAT3 in colon cancer cells

**Figure 3:**
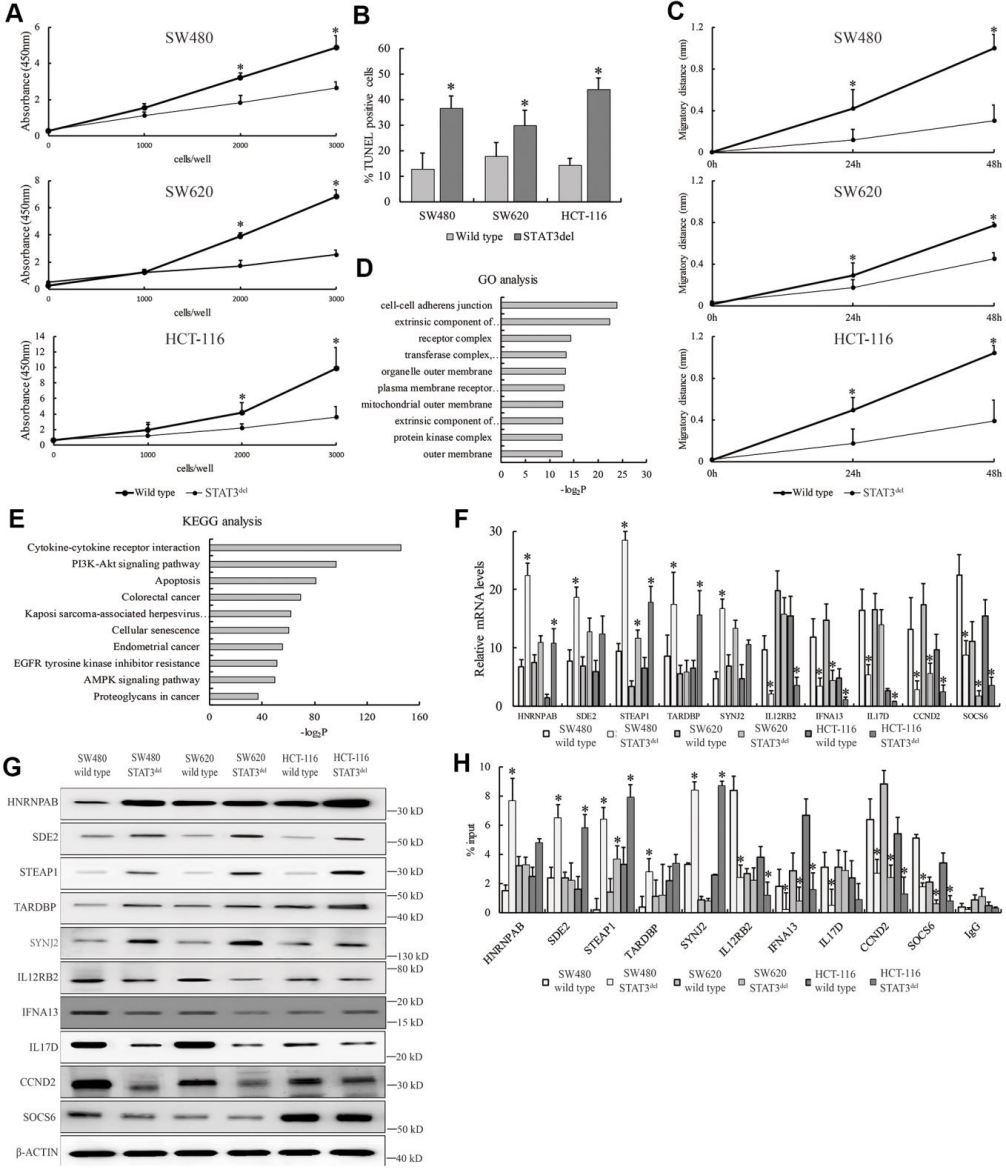
**STAT3^del^ reduces *in vitro* growth, survival and migration of colon cancer cells.** (**A**) CCK8 assay results show the proliferation rate of SW480, SW620 and HCT-116 cells with wild type STAT3 or STAT3^del^. (**B**) TUNEL assay results show the apoptotic rate of SW480, SW620 and HCT-116 cells with wild type STAT3 or STAT3^del^. (**C**) Wound healing assay results show the migration rate of SW480, SW620 and HCT-116 cells with wild type STAT3 or STAT3^del^. * denotes *p* <0.05. Each experiment was performed with three replicates. (**D**, **E**) The top ten enriched GO terms related to biological process (**D**) and KEGG signaling pathways (**E**) based on the genes associated with differential peaks. (**F**) The mRNA and (**G**) protein levels of several genes with differential STAT3 binding ability in SW480 cells with wild type STAT3 or STAT3^del^. (**H**) STAT3 occupancy in genes with differential STAT3 binding ability in the SW480, SW620 and HCT116 cells with wild type STAT3 or STAT3^del^. * denotes *p* < 0.05. Each experiment was performed with three replicates.

Since hyper-activation of the JAK/STAT3 signaling pathway promotes survival of cancer cells, we investigated the proliferation, migration, and survival status of SW480 cells with STAT3^del^ in comparison with the wild-type SW480 cells. MTT, wound healing and TUNEL assays demonstrated that proliferation, migration and survival of SW480 cells with STAT3^del^ was significantly reduced compared to the wild type SW480 cells (Figure 3A–3C). The top ten Gene Ontology (GO) terms related to biological function (BF) and Kyoto Encyclopedia of Genes and Genomes (KEGG) pathways that are differentially activated in SW480 cells with STAT3^del^ based on the ChIP-seq data are shown in Figure 3D, 3E. We then used qRT-PCR and western blotting analyses to determine the mRNA and protein expression levels of the top five genes showed enhanced STAT3^del^ binding, namely, HNRNPAB, SDE2, STEAP1, TARDBP and SYNJ2, as well as top five genes that showed reduced STAT3^del^ binding, namely, IL12RB2, IFNA13, IL17D, CCND2 and SOCS6 in the SW480, SW620 and HCT116 cells with STAT3 or STAT3^del^. Majority of the target genes with enhanced binding of STAT3^del^ showed significantly higher expression, whereas majority those that showed reduced STAT3^del^ binding demonstrated significantly reduced expression in the three colon cancer cells with STAT3^del^ compared to those with wild-type STAT3 (Figure 3F, 3G). STEAP1 plays an integral role in the cell junctions and performs a tumor suppressor function in endometrial carcinomas [13], breast cancers [14] and gastric cancer [15]. STEAP1 was up-regulated in the colon cancer cells with STAT3^del^ compared to those with the wild type STAT3 (Figure 3F, 3G). We also determined the binding sites of STAT3^del^ in the promoter regions of the target genes in the mutant SW480, SW620 and HCT-116 cells using ChIP-qPCR (Figure 3H). Overall, STAT3^del^ enrichment was consistent with the expression of the target genes (Figure 3F, 3G). Overall, our results demonstrate that altered genome-wide binding of STAT3^del^ suppresses tumor growth and progression. Hence, mutations in the DNA-binding domain of STAT3 may be potentially useful in determining cancer diagnosis.

## DISCUSSION

Several studies have shown that JAK/STAT3 signaling pathway promotes colorectal carcinogenesis [16]. The SH2 domain of STAT3 is phosphorylated by JAKs that are activated by multiple ligands such as interleukins [17], EGF [18], HGF [19], FGF [20], and VEGF [21]. The activated STAT3 dimerizes, translocates to the nucleus and binds to its target gene sequences through the DNA-binding domain [22, 23]. Hyperactivation of STAT3 enhances the expression of its target genes, which promote tumor cell migration and proliferation. However, mutations in the *STAT3* gene, especially in its DNA-binding domain, diminish its ability to promote gene transcription despite normal activation of the JAK/STAT3 signaling pathway, and thereby represent a potential avenue for cancer therapy. In this study, we demonstrate that the natural mutations in the DNA-binding domain of STAT3 are associated with reduced malignancy of several cancers (Figure 1). Moreover, mutations in the SH2 and other regions of STAT3 are associated with increased tumor malignancy (Figure 1A) because they induce hyper-activation of the JAK/STAT pathway and resistance to dephosphorylation of STAT3 [24]. However, the sample size of human cancer samples containing STAT3 mutations is low and hence this conclusion needs to be supported by further evidence.

In this study, we investigated the role of mutations in the DNA-binding domain of STAT3 in carcinogenesis. However, deleting the entire DNA-binding domain of STAT3 is not the best option because it could be toxic or completely abolish the JAK/STAT3 pathway signaling. Therefore, we deleted short sequence that represents a hot spot mutant region in the DNA-binding domain involving amino acid residues between 400 and 411 (NNGSLSAEFKHL). We observed that this region was one of the most frequently mutated sequences in epithelial cancers according to TCGA database analysis. We then used ChIP-seq analysis to evaluate the differential binding patterns of STAT3^del^ and wild-type STAT3 in multiple colon cancer cells. Overall, we observed reduced STAT3^del^ binding to the known target gene sequences compared to the wild-type (Figure 2D, 2E). We also observed that STAT3^del^ bound to new DNA sequences compared to the wild-type STAT3 (Figure 2F–2H). Based on the STAT3/DNA complex (PDB code 1BG1) [25], we observed that the amino acid residues from 430 to 470 formed the core sequence that directly interacted with the DNA. However, “NNGSLSAEFKHL” constitutes a β-sheet, which acts as a scaffold to support and maintain STAT3 close to the DNA. Therefore, deleting this sequence may reduce the binding affinity between STAT3 and DNA, but these data require analyzing the crystal structure of STAT3^del^, which is beyond the scope of our study.

Consistent with the *in vivo* data in human cancer samples from the TCGA database, we demonstrate that STAT3^del^ reduces growth, progression, and survival of SW480 cells (Figure 3A–3C). We also demonstrate that the higher expression of multiple genes such as *SDE2*, *STEAP1*, and *SYNJ2* is associated with enhanced binding of STAT3^del^ to their gene sequences including promoters (Figure 2G, 3F–3H). STEAP1 is a tumor suppressor gene in colon, breast and gastric cancers, but acts as an oncogene in prostate cancer [26, 27]. Our data demonstrates that STAT3^del^ aberrantly regulates several genes, which need to be evaluated as potential risk factors for colon cancer diagnosis.

Taken together, our data demonstrates that deletion of the hotspot mutation region in the DNA-binding domain of STAT3 reduces colon cancer cell growth and progression because of genome-wide changes in the transcription of STAT3-target genes. Therefore, we postulate that mutations in the DNA-binding domain of *STAT3* or the novel downstream genes that are upregulated by the mutant STAT3 are potential biomarkers for colon cancer diagnosis.

## MATERIALS AND METHODS

### TCGA data mining

We compared the correlation between STAT3 mutations and tumor malignancy, overall survival rate, and STAT3 expression in 10528 cases belonging to 32 types of cancer from the TCGA database [28]. The data was downloaded from the c-Bioportal (https://www.cbioportal.org/) [3, 29].

### Generation of colon cancer cell lines with STAT3 deletion

We obtained SW480, SW620 and HCT116 colon cancer cell lines from the Type Culture Collection of the Chinese Academy of Sciences (Beijing, China) and cultured them in DMEM medium with 10% FBS (Thermo Fisher Scientific, USA) in a humidified incubator at 37° C and 5% CO_2_. To generate a cell line with a specific *STAT3* deletion, we cloned gRNA oligonucleotides (http://crispr.mit.edu): 5’-CCGACCCGATAGGCTGCAAGG-3’ and 5’- AGGCACCGTCGAGACGCGG -3’ into px330 plasmid (Cat. No. 73131; Addgene), and transfected the recombinant plasmids into the colon cancer cells using lipofectamine 3000 (Thermo Fisher Scientific, Waltham, USA). The positive clones were screened using DMEM medium containing 10 μg/ml puromycin. The partial STAT3 exon 10 (1198-1233 bp) containing the deleted sequence was confirmed by Sanger sequencing. The wild-type and mutant colon cancer cells were treated with 300 nM TG101348 (MedChemExpress, Monmouth Junction, USA), a JAK2 inhibitor, for 48 h [30].

### Chromatin immunoprecipitation (ChIP) assay

The ChIP assay was performed as previously described [31]. In brief, whole cell lysates were sonicated to break up the genomic DNA into 200-500 bp fragments. Then, 10% of the sonicated whole cell lysates were saved as input and the remaining were incubated with 1 μg IP grade STAT3 antibody (#4904, CST, Danvers, USA) overnight at 4° C. This was followed by incubating for 2 h with Protein A beads (Thermo Fisher Scientific) at 37° C to pull down STAT3-bound DNA fragments. We then isolated the DNA fragments from the pull-down by ethanol precipitation and performed quantitative PCR assay (see the qPCR method section for details). For high throughput sequencing, we added 3’-dA overhang to the STAT3 enriched or input DNA and ligated them to the adapter to build a DNA library. DNA library with ligated adapters were isolated based on appropriate size for sequencing using the Illumina Hiseq2000 platform. The raw sequence reads of input and IP were trimmed based on adaptors and low quality reads were filtered out using Cutadapt (v1.9.1) and Trimmomatic (v0.35) [32]. The quality of the clean reads was checked using Fastqc [33]. The clean reads were mapped to the human genome (assembly hg38) using the Bowtie 2 (v2.2.6) algorithm [34], and peak calling (*p*<0.01) was performed using MACS 2 (v2.1.1) [35]. The genes that bound differentially to STAT3^del^ relative to wild-type STAT3 were analyzed based on FDR value less than 0.05 and annotated using DiffBind [36]. *De novo* motifs were analyzed using R language and MEME [37–40]. The relevant peaks on the genomic loci were visualized using the Integrative Genomics Viewer (IGV). Gene ontology (GO) analysis was used to determine the biological function of genes associated with the differential peaks [40]. The ChIP sequencing data was submitted to the SRA database and registered with the accession number E-MTAB-8417.

### Quantitative PCR

We extracted total RNA from SW480 cells using TRIZOL (Takara, Dalian City, China). We then reverse transcribed equal amounts of RNA samples to generate cDNA using QuantiTect Reverse Transcription Kit (Qiagen, Germany). We then performed quantitative PCR (qPCR) using the QuantStudio 3 Real-time PCR system (Thermo Fisher Scientific). The qPCR protocol included initial denaturation at 95° C for 30 s followed by 40 cycles at 95° C for 5 s and annealing for 10 s at 72° C. The final annealing was at 72° C for 30 s. The relative expression was calculated from the Ct values by using the 2^-ΔΔCt^ method. β-actin was used as the internal control. All primers used in this study are listed in Supplementary Table 1.

### Western blotting

Total cellular protein extracts were prepared using the RIPA buffer (Beyotime, Shanghai, China) and the protein concentration was determined using the bicinchoninic acid (BCA) assay. Equal amounts of protein samples were resolved by SDS-PAGE and transferred to the PVDF membranes. The membranes were then blocked for 30 mins with 5% skimmed milk in 1X TBST buffer and then incubated overnight at 4° C with primary antibodies against JAK2 (#3230, CST, 1:2000), p-JAK2 (#3771, CST, 1:500), STAT3 (#4904, CST, 1:2000), p-STAT3 (#9145, CST, 1:500), HNRNPAB (CSB-PA010604LA01HU, CUSABIO, 1:3000), SDE2 (#491314, US Biological, 1:2000), STEAP1 (#88677S, CST, 1:2500), TARDBP (#89789, CST, 1:4000), SYNJ2 (#AP19970c, ABCEPTA, 1:500), IL12RB2 (PA5-21479, Thermo Fisher, 1:5000), IFNA13 (MA5-24415, Thermo Fisher, 1:5000), IL17D (MA5-23946, Thermo Fisher, 1:5000), CCND2 (MA5-12731, Thermo Fisher, 1:2000), SOCS6 (PA5-50263, Thermo Fisher, 1:2500), and β-actin (#3700, 1:5000). Then, the blots were incubated with the horseradish peroxidase (HRP)-conjugated anti-mouse or anti-rabbit secondary antibodies (1: 10000, Beyotime) at room temperature for 1 h. β-actin was used as the loading control. The blots were developed using ECL (Pierce, USA), photographed using Image Lab (Bio-Rad) and analyzed using Image J.

### CCK-8 cell proliferation assay

SW480 cells were cultured in a 96 well plate until they reached 80% density. Then, 10 μl CCK-8 solution (Solarbio, China) was added to all wells and the cells were further incubated for 1 h. Then, the absorption values were measured at 450 nm using the Multiskan FC microplate reader (Thermo Fisher Scientific, USA) and the IC_50_ value was calculated using the regression equation. The cell proliferation rates were determined based on the IC_50_ value of each sample.

### TUNEL assay

We transferred 100 μl of the 1-2×10^7^ cells/mL in PBS into a 96-well plate and fixed the cells on ice using 2% formaldehyde for 15 min. The cells were then washed by PBS, then incubated 1×10^5^ cells in 50 μl TdT equilibration buffer (Beyotime) at 37° C for 10 min with occasional gentle mixing. Then, after washing the cells twice with PBS, the cells were incubated with 100 μl TdT staining buffer at room temperature for 30 min in the dark. The cells were then mounted on coverslips using the mounting medium (Sigma-Aldrich, San Francisco, USA) and imaged under a DM2000 fluorescence microscope (Leica Biosystems, Wetzlar, Germany).

### Scratch wound healing assay

We cultured 1×10^5^ colon cancer cells in 6-well plates overnight to form a uniform monolayer. Then, the monolayer was scratched with a sterile tip to generate a wound and the floating cells were removed by washing twice with PBS. Then, the cells were cultured in DMEM medium without FBS for 48 h. Images were captured at 0 (T0), 24 (T24), and 48 (T48) h. The edge distance and the scratch area was measured at all time points using Image J. The cell migration distance was calculated as Area at zero time (T0)-Area at 24h or 48h (Tt).

### Statistical analysis

The experimental data was analyzed using the SPSS 20.0 software. The chi-square (χ^2^) test was used to analyze the correlation between *STAT3* mutations in different domains and tumor malignancy. Kaplan-Meier survival curve analysis was used to evaluate the correlation between STAT3 mutations in different domains and the survival rate of cancer patients. Student’s t-test was used to compare the differences between the wild type and mutant STAT3 groups. *P* < 0.05 was considered statistically significant.

## Supplementary Material

Supplementary Tables

## References

[1] Favoriti P, Carbone G, Greco M, Pirozzi F, Pirozzi RE, Corcione F. Worldwide burden of colorectal cancer: a review. Updates Surg. 2016; 68:7–11. 10.1007/s13304-016-0359-y 27067591

[2] Hadjipetrou A, Anyfantakis D, Galanakis CG, Kastanakis M, Kastanakis S. Colorectal cancer, screening and primary care: a mini literature review. World J Gastroenterol. 2017; 23:6049–58. 10.3748/wjg.v23.i33.6049 28970720 PMC5597496

[3] Gao J, Aksoy BA, Dogrusoz U, Dresdner G, Gross B, Sumer SO, Sun Y, Jacobsen A, Sinha R, Larsson E, Cerami E, Sander C, Schultz N. Integrative analysis of complex cancer genomics and clinical profiles using the cBioPortal. Sci Signal. 2013; 6:pl1. 10.1126/scisignal.2004088 23550210 PMC4160307

[4] Banerjee S, Biehl A, Gadina M, Hasni S, Schwartz DM. JAK-STAT signaling as a target for inflammatory and autoimmune diseases: current and future prospects. Drugs. 2017; 77:521–46. 10.1007/s40265-017-0701-9 28255960 PMC7102286

[5] Morris R, Kershaw NJ, Babon JJ. The molecular details of cytokine signaling via the JAK/STAT pathway. Protein Sci. 2018; 27:1984–2009. 10.1002/pro.3519 30267440 PMC6237706

[6] Hüntelmann B, Staab J, Herrmann-Lingen C, Meyer T. A conserved motif in the linker domain of STAT1 transcription factor is required for both recognition and release from high-affinity DNA-binding sites. PLoS One. 2014; 9:e97633. 10.1371/journal.pone.0097633 24847715 PMC4029728

[7] Kang K, Robinson GW, Hennighausen L. Comprehensive meta-analysis of signal transducers and activators of transcription (STAT) genomic binding patterns discerns cell-specific cis-regulatory modules. BMC Genomics. 2013; 14:4. 10.1186/1471-2164-14-4 23324445 PMC3564941

[8] Husby J, Todd AK, Haider SM, Zinzalla G, Thurston DE, Neidle S. Molecular dynamics studies of the STAT3 homodimer:DNA complex: relationships between STAT3 mutations and protein-DNA recognition. J Chem Inf Model. 2012; 52:1179–92. 10.1021/ci200625q 22500887

[9] Mohr A, Fahrenkamp D, Rinis N, Müller-Newen G. Dominant-negative activity of the STAT3-Y705F mutant depends on the N-terminal domain. Cell Commun Signal. 2013; 11:83. 10.1186/1478-811X-11-83 24192293 PMC3833267

[10] Yadav A, Kumar B, Datta J, Teknos TN, Kumar P. IL-6 promotes head and neck tumor metastasis by inducing epithelial-mesenchymal transition via the JAK-STAT3-SNAIL signaling pathway. Mol Cancer Res. 2011; 9:1658–67. 10.1158/1541-7786.MCR-11-0271 21976712 PMC3243808

[11] Wei KL, Chou JL, Chen YC, Jin H, Chuang YM, Wu CS, Chan MW. Methylomics analysis identifies a putative STAT3 target, SPG20, as a noninvasive epigenetic biomarker for early detection of gastric cancer. PLoS One. 2019; 14:e0218338. 10.1371/journal.pone.0218338 31194837 PMC6564691

[12] Liu M, Wilson NO, Hibbert JM, Stiles JK. STAT3 regulates MMP3 in heme-induced endothelial cell apoptosis. PLoS One. 2013; 8:e71366. 10.1371/journal.pone.0071366 23967200 PMC3742773

[13] Sun J, Ji G, Xie J, Jiao Z, Zhang H, Chen J. Six-transmembrane epithelial antigen of the prostate 1 is associated with tumor invasion and migration in endometrial carcinomas. J Cell Biochem. 2019. [Epub ahead of print]. 10.1002/jcb.28393 30714206

[14] Xie J, Yang Y, Sun J, Jiao Z, Zhang H, Chen J. STEAP1 inhibits breast cancer metastasis and is associated with epithelial-mesenchymal transition procession. Clin Breast Cancer. 2019; 19:e195–207. 10.1016/j.clbc.2018.08.010 30253922

[15] Wu YY, Jiang JN, Fang XD, Ji FJ. STEAP1 regulates tumorigenesis and chemoresistance during peritoneal metastasis of gastric cancer. Front Physiol. 2018; 9:1132. 10.3389/fphys.2018.01132 30246786 PMC6110897

[16] Wang SW, Sun YM. The IL-6/JAK/STAT3 pathway: potential therapeutic strategies in treating colorectal cancer (review). Int J Oncol. 2014; 44:1032–40. 10.3892/ijo.2014.2259 24430672

[17] Johnson DE, O’Keefe RA, Grandis JR. Targeting the IL-6/JAK/STAT3 signalling axis in cancer. Nat Rev Clin Oncol. 2018; 15:234–48. 10.1038/nrclinonc.2018.8 29405201 PMC5858971

[18] Huang Q, Li S, Zhang L, Qiao X, Zhang Y, Zhao X, Xiao G, Li Z. CAPE- *p* NO_2_ inhibited the growth and metastasis of triple-negative breast cancer via the EGFR/STAT3/Akt/E-Cadherin signaling pathway. Front Oncol. 2019; 9:461. 10.3389/fonc.2019.00461 31214503 PMC6558049

[19] Zakaria S, Helmy MW, Salahuddin A, Omran G. Chemopreventive and antitumor effects of benzyl isothiocynate on HCC models: a possible role of HGF /pAkt/ STAT3 axis and VEGF. Biomed Pharmacother. 2018; 108:65–75. 10.1016/j.biopha.2018.09.016 30216802

[20] Dong Z, Santeford A, Ban N, Lee TJ, Smith C, Ornitz DM, Apte RS. FGF2-induced STAT3 activation regulates pathologic neovascularization. Exp Eye Res. 2019; 187:107775. 10.1016/j.exer.2019.107775 31449793 PMC6759401

[21] Zhao D, Pan C, Sun J, Gilbert C, Drews-Elger K, Azzam DJ, Picon-Ruiz M, Kim M, Ullmer W, El-Ashry D, Creighton CJ, Slingerland JM. VEGF drives cancer-initiating stem cells through VEGFR-2/Stat3 signaling to upregulate Myc and Sox2. Oncogene. 2015; 34:3107–19. 10.1038/onc.2014.257 25151964

[22] Akira S, Nishio Y, Inoue M, Wang XJ, Wei S, Matsusaka T, Yoshida K, Sudo T, Naruto M, Kishimoto T. Molecular cloning of APRF, a novel IFN-stimulated gene factor 3 p91-related transcription factor involved in the gp130-mediated signaling pathway. Cell. 1994; 77:63–71. 10.1016/0092-8674(94)90235-6 7512451

[23] Guanizo AC, Fernando CD, Garama DJ, Gough DJ. STAT3: a multifaceted oncoprotein. Growth Factors. 2018; 36:1–14. 10.1080/08977194.2018.1473393 29873274

[24] de Araujo ED, Erdogan F, Neubauer HA, Meneksedag-Erol D, Manaswiyoungkul P, Eram MS, Seo HS, Qadree AK, Israelian J, Orlova A, Suske T, Pham HT, Boersma A, et al. Structural and functional consequences of the STAT5B^N642H^ driver mutation. Nat Commun. 2019; 10:2517. 10.1038/s41467-019-10422-7 31175292 PMC6555848

[25] Ren Z, Mao X, Mertens C, Krishnaraj R, Qin J, Mandal PK, Romanowski MJ, McMurray JS, Chen X. Crystal structure of unphosphorylated STAT3 core fragment. Biochem Biophys Res Commun. 2008; 374:1–5. 10.1016/j.bbrc.2008.04.049 18433722

[26] Barroca-Ferreira J, Pais JP, Santos MM, Goncalves AM, Gomes IM, Sousa I, Rocha SM, Passarinha LA, Maia CJ. Targeting STEAP1 protein in human cancer: current trends and future challenges. Curr Cancer Drug Targets. 2018; 18:222–30. 10.2174/1568009617666170427103732 28460619

[27] Gomes IM, Rocha SM, Gaspar C, Alvelos MI, Santos CR, Socorro S, Maia CJ. Knockdown of STEAP1 inhibits cell growth and induces apoptosis in LNCaP prostate cancer cells counteracting the effect of androgens. Med Oncol. 2018; 35:40. 10.1007/s12032-018-1100-0 29464393

[28] Weinstein JN, Collisson EA, Mills GB, Shaw KR, Ozenberger BA, Ellrott K, Shmulevich I, Sander C, Stuart JM, and Cancer Genome Atlas Research Network. The cancer genome atlas pan-cancer analysis project. Nat Genet. 2013; 45:1113–20. 10.1038/ng.2764 24071849 PMC3919969

[29] Cerami E, Gao J, Dogrusoz U, Gross BE, Sumer SO, Aksoy BA, Jacobsen A, Byrne CJ, Heuer ML, Larsson E, Antipin Y, Reva B, Goldberg AP, et al. The cBio cancer genomics portal: an open platform for exploring multidimensional cancer genomics data. Cancer Discov. 2012; 2:401–04. 10.1158/2159-8290.CD-12-0095 22588877 PMC3956037

[30] Ikeda S, Okamoto T, Okano S, Umemoto Y, Tagawa T, Morodomi Y, Kohno M, Shimamatsu S, Kitahara H, Suzuki Y, Fujishita T, Maehara Y. PD-L1 is upregulated by simultaneous amplification of the PD-L1 and JAK2 genes in non-small cell lung cancer. J Thorac Oncol. 2016; 11:62–71. 10.1016/j.jtho.2015.09.010 26762740

[31] Fei P, Wang W, Kim SH, Wang S, Burns TF, Sax JK, Buzzai M, Dicker DT, McKenna WG, Bernhard EJ, El-Deiry WS. Bnip3L is induced by p53 under hypoxia, and its knockdown promotes tumor growth. Cancer Cell. 2004; 6:597–609. 10.1016/j.ccr.2004.10.012 15607964

[32] Bolger AM, Lohse M, Usadel B. Trimmomatic: a flexible trimmer for illumina sequence data. Bioinformatics. 2014; 30:2114–20. 10.1093/bioinformatics/btu170 24695404 PMC4103590

[33] Andrews S. Babraham Bioinformatics -FastQC A Quality Control tool for High Throughput Sequence Data. 2013.

[34] Langmead B, Salzberg SL. Fast gapped-read alignment with Bowtie 2. Nat Methods. 2012; 9:357–59. 10.1038/nmeth.1923 22388286 PMC3322381

[35] Zhang Y, Liu T, Meyer CA, Eeckhoute J, Johnson DS, Bernstein BE, Nusbaum C, Myers RM, Brown M, Li W, Liu XS. Model-based analysis of ChIP-Seq (MACS). Genome Biol. 2008; 9:R137. 10.1186/gb-2008-9-9-r137 18798982 PMC2592715

[36] Ghali RM, Al-Mutawa MA, Al-Ansari AK, Zaied S, Bhiri H, Mahjoub T, Almawi WY. Differential association of ESR1 and ESR2 gene variants with the risk of breast cancer and associated features: a case-control study. Gene. 2018; 651:194–99. 10.1016/j.gene.2018.02.011 29414691

[37] Bailey TL, Johnson J, Grant CE, Noble WS. The MEME suite. Nucleic Acids Res. 2015; 43:W39–49. 10.1093/nar/gkv416 25953851 PMC4489269

[38] Li H, Su X, Gallegos J, Lu Y, Ji Y, Molldrem JJ, Liang S. dsPIG: a tool to predict imprinted genes from the deep sequencing of whole transcriptomes. BMC Bioinformatics. 2012; 13:271. 10.1186/1471-2105-13-271 23083219 PMC3497615

[39] Kong H, Tong P, Zhao X, Sun J, Li H. CAsubtype: an R package to identify gene sets predictive of cancer subtypes and clinical outcomes. Interdiscip Sci. 2018; 10:169–75. 10.1007/s12539-016-0198-z 28110480

[40] Subramanian A, Tamayo P, Mootha VK, Mukherjee S, Ebert BL, Gillette MA, Paulovich A, Pomeroy SL, Golub TR, Lander ES, Mesirov JP. Gene set enrichment analysis: a knowledge-based approach for interpreting genome-wide expression profiles. Proc Natl Acad Sci USA. 2005; 102:15545–50. 10.1073/pnas.0506580102 16199517 PMC1239896

