# Supplementary material for: A short deletion in the DNA-binding domain of STAT3 suppresses growth and progression of colon cancer cells
Source: Aging (Albany NY). 2021 Feb 1;13(4):5185–96. doi: 10.18632/aging.202439 (PMC7950243; doi:10.18632/aging.202439)
Supplement: Supplementary Tables [file aging-13-202439-s001.pdf]

SUPPLEMENTARY TABLES

Supplementary Table 1. All the primers used in this study were listed in this table.

Symbol	Sequence	Tm (° C)
STAT3 for identification and detection	TCAGGTTGCTGGTCA<br>GGTCAGCATGTTGTACC	56
Snail for ChIP-assay	TCGGCAGCTGAACCGA<br>GCCGATTACCGTGCAG	60
Spg20 for ChIP-assay	GCCGATTTGACCGAG<br>CGTCCGTGAATTGACC	58
Mmp3 for ChIP-assay	GGCCTCGGCGCAGCCGA<br>GACCGAGCGCTGCAG	58
HNRNPAB for ChIP-assay	TGGCCGAGAGGACGG<br>GGGTTACACCTTCGAA	62
SDE2 for ChIP-assay	AATGCGGCTAGCAGGCC<br>GAGGCCCCGGAGCCAATGG	54
STEAP1 for ChIP-assay	GAGCCGTTGCAGCTG<br>GAGACGTGCACATGG	60
TARDBP for ChIP-assay	TTTCTTATCTTTGGCCA<br>GGATTCGTTCACGTT	58
SYNJ2 for ChIP-assay	TTGTGCAGTACCCAGGTG<br>TAGTTATCCGATTGA	56
IL12RB2 for ChIP-assay	ATGCTGTGCACACACT<br>CATTGCTGATCACACA	58
IFNA13 for ChIP-assay	ACATGCTGTGGCACAGGC<br>GGGGTGCGGCTAACCGGTT	60
IL17D for ChIP-assay	TTGGTTACCCAAAC<br>CAACACACTTGTGCAC	60
CCND2 for ChIP-assay	CACACTGTTGGTTTTCACA<br>CACCGGCTCCTTCCGACC	58
SOCS6 for ChIP-assay	CCACTTGGCACCACA<br>TTGGTTCACACGTG	60
HNRNPAB for qPCR	TTGGTGCTGATGTGTGAC<br>GATGACACGTGAGATG	60
SDE2 for qPCR	GATGATGGAGTGGG<br>GGTGTGTCCTGCTACA	58
STEAP1 for qPCR	CCAATCCTGGTCAT<br>GAGACACATAAATCTC	58
TARDBP for qPCR	TATCTACTTGGCTATCAGA<br>CACGTGAGTGGACCAC	56
SYNJ2 for qPCR	TGATGGCCACAGTG<br>CACAGGGGGTGCATGAAGTT	56
IL12RB2 for qPCR	CAAATTGGTAGCTGACA<br>ATTGGACAATTGGACATGA	55
IFNA13 for qPCR	CCAATCCTGGTCAT<br>GAGACACATAAATCTC	58
IL17D for qPCR	AACCTTTCATAGGGAC<br>GGAGCCACGGTGGAC	58

CCND2 for qPCR	GGAGGACCCATCTACACGG<br>GAGGGCCATCTGGCACA	58
SOCS6 for qPCR	GAGCAGGAGAGACAGT<br>GACCACACGGGACCCATTT	56
β-actin	CAGGGCGTGATGGTGGGCA<br>CAAACATCATCTGGGTCATCTTCTC	58

Supplementary Table 2. The basic information of the sequencing data.

Sample	MT1_Input	MT2_Input	MT1	MT2	WT1_Input	WT2_Input	WT1	WT2
Total Raw Reads	20347878	21145236	20050877	20079109	20154533	21352412	20234033	21431912
Total Raw Bases	1546438728	1607037936	1523866652	1526012284	1531744508	1622783312	1537786508	1628825312
Total Clean Reads	20229383	21027590	19962725	19999408	20041558	21224271	20159353	21147387
Total Clean Bases	1525944696	1586740803	1507071661	1510655073	1512346277	1600610000	1523162485	1585258639
Mapped Reads	20008881	20798048	19550649	19583796	19822067	20991514	19453838	20407766
Mapped Ratio	98.91%	98.91%	97.94%	97.92%	98.90%	98.90%	96.50%	96.50%
Unique Mapped Reads	15657282	16286655	15430889	15470657	15522511	16426580	15524317	16207577
Unique Mapped Ratio	77.40%	77.45%	77.30%	77.36%	77.45%	77.40%	77.01%	76.64%
